# Epigenetic Changes of Lentiviral Transgenes in Porcine Stem Cells Derived from Embryonic Origin

**DOI:** 10.1371/journal.pone.0072184

**Published:** 2013-08-19

**Authors:** Kwang-Hwan Choi, Jin-Kyu Park, Hye-Sun Kim, Kyung-Jun Uh, Dong-Chan Son, Chang-Kyu Lee

**Affiliations:** Department of Agricultural Biotechnology, Animal Biotechnology Major, and Research Institute for Agriculture and Life Science, Seoul National University, Seoul, Korea; Indian Institute of Toxicology Reserach, India

## Abstract

Because of the physiological and immunological similarities that exist between pigs and humans, porcine pluripotent cell lines have been identified as important candidates for preliminary studies on human disease as well as a source for generating transgenic animals. Therefore, the establishment and characterization of porcine embryonic stem cells (pESCs), along with the generation of stable transgenic cell lines, is essential. In this study, we attempted to efficiently introduce transgenes into Epiblast stem cell (EpiSC)-like pESCs. Consequently, a pluripotent cell line could be derived from a porcine-hatched blastocyst. Enhanced green fluorescent protein (EGFP) was successfully introduced into the cells via lentiviral vectors under various multiplicities of infection, with pluripotency and differentiation potential unaffected after transfection. However, EGFP expression gradually declined during extended culture. This silencing effect was recovered by *in vitro* differentiation and treatment with 5-azadeoxycytidine. This phenomenon was related to DNA methylation as determined by bisulfite sequencing. In conclusion, we were able to successfully derive EpiSC-like pESCs and introduce transgenes into these cells using lentiviral vectors. This cell line could potentially be used as a donor cell source for transgenic pigs and may be a useful tool for studies involving EpiSC-like pESCs as well as aid in the understanding of the epigenetic regulation of transgenes.

## Introduction

Over the last three decades, the establishment of pluripotent cell lines from preimplantation mouse embryos has been considered to be one of the biggest events in developmental biology [1], [2]. These cells, known as embryonic stem cells, have *in vivo* and *in vitro* differentiation potentials into three germ layers and can proliferate infinitely. Recently, mouse epiblast stem cells (EpiSCs) and induced pluripotent stem cells (iPSCs) were derived from postimplantation embryos and somatic cells, respectively [3], [4]. These pluripotent cells are divided into “naïve” and “primed” states by their pluripotent status [5]. In permissive lines, pluripotent cells can be derived from embryos in both states. However, in nonpermissive lines such as human and pig, cells are only derived into the “primed” state, such as epiblast stem cells, if no additional treatment such as genetic manipulation and chemicals are performed [6]–[8].

Pluripotent cells are expected to be used as cell therapeutic material in degenerative disorders, and in domestic animals, as cell sources for generating transgenic animals and xenotransplantation [9]. In particular, in transgenic animal and xenotransplantation applications, pigs have been identified as an ideal animal model because of similarities between humans and pigs in physiological and immunological features, as well as organ size [10], [11]. Therefore, many research groups have attempted to create transgenic pigs to produce pharmaceutical proteins and in xenotransplantation [12], [13]. In addition, although authentic porcine embryonic stem cells (pESCs) have not yet been established, the characterization of pESCs, along with the generation of stable transgenic cell lines, has been studied for a long time [8], [14]–[18].

To achieve these goals, genetic manipulation via transgenic technologies has been required in stem cell research. Transgenic stem cells using the homologous recombination technique were first reported in mouse embryonic stem cells by Thomas & Capecchi in 1987 [19]. Subsequently, researchers have successfully delivered transgenes into pluripotent stem cells using several methods, including electroporation [20], liposomal [21] and viral vectors [22], [23], and nucleofection [24]. However, stably introducing transgenes in these cells has proven difficult because of the low efficiency and cytotoxic side effects. The delivery of transgenes using viral vectors, which are stably expressed, is considered the most useful tool for inducing low cytotoxicity and inserting transgenes into the host genome [25]. Moreover, lentiviral vectors belonging to retroviral families are able to infect several types of cells, as well as nondividing cells [26], [27].

Transgenesis in porcine embryonic stem cells was first reported by Yang *et al*. (2009) [17]. In contrast to other reports using somatic cell nuclear transfer (SCNT) with transgenic donor cells [28], [29], the transgene [humanized renilla green fluorescent protein (hrGFP)] was directly delivered into pESCs via electroporation. Stably hrGFP-expressing porcine pluripotent cell lines were successfully established by introducing plasmid vectors via electroporation. Transgenic porcine embryonic germ cell lines were reported by Rui *et al*. (2006) [30]. In this study enhanced green fluorescent protein (EGFP) transgenes were introduced into cells with a liposomal vector. In other studies involving mouse embryonic stem cells, GFP-expressing lines were successfully established using viral vectors [31], [32]. However, GFP expression gradually decreased during passaging in mouse embryonic stem cells due to DNA methylation. In a similar case of transgenic animal production by lentiviral transduction, transgenes were silenced by DNA methylation in specific cell types [33], [34].

Recently, we derived an epiblast stem cell-like pESC lines (EpiSC-like pESCs) from hatched blastocysts, which showed EpiSC-like features [8]. As we established these porcine pluripotent stem cell lines, we attempted to generate a transgenic pluripotent cell line. As mentioned above, transgenesis in porcine pluripotent cells is essential for applications such as the production of transgenic pigs and analysis of gene functions. Moreover it is important to characterize and optimize an efficient transfection system. The main purpose of this study was to successfully introduce transgenes into EpiSC-like pESCs using lentiviral vectors, and to optimize these viral infection conditions. Additionally, we evaluated the relationship between transgene expression and changes in the DNA methylation status of the inserted lentiviral transgene, particularly in the promoter regions of undifferentiated and differentiated EpiSC-like pESCs. Consequently, transgenes were successfully introduced into the cells via lentiviral vectors under various multiplicities of infection. Furthermore, it was confirmed that the expression of inserted lentiviral transgenes was controlled by DNA methylation. This cell line could potentially be used as a donor cell source for transgenic pigs and may be a useful tool for studies of gene functions involving EpiSC-like pESCs.

## Materials and Methods

### Animal Welfare

The care and experimental use of pigs and mice was approved by the Institute of Laboratory Animal Resources, Seoul National University (SNU-110509-1).

### Isolation and Culture of Epiblast Stem Cell-like Porcine Embryonic Stem Cells

EpiSC-like pESCs were derived from *in vitro*-produced blastocysts. *In vitro*-produced and hatched blastocysts were seeded on feeder cells composed of mitotically inactivated mouse embryonic fibroblasts (MEFs) according to our previously studies [8], [16]. After 5–7 days, primary colonies of embryonic stem cells were observed and cultured for approximately 7–10 days longer. Fully expanded colonies were mechanically dissociated using pulled-glass pipettes and transferred onto new feeder cells for subculture.

EpiSC-like pESCs were cultured in porcine embryonic stem cell media (PESM). PESM consisted of 1∶1 mixture of Dulbecco’s modified Eagle’s medium (DMEM, low glucose) and Ham’s F10 media containing 15% fetal bovine serum (FBS; collected and processed in the USA), 2 mM glutamax, 0.1 mM β-mercaptoethanol, 1× MEM nonessential amino acids, and 1× antibiotic–antimycotic (all from Gibco, USA). To support pluripotency and self-renewal, the embryonic stem cells were cultured in PESM with the following cytokines: 40 ng/ml human recombinant stem cell factor (hrSCF; R&D Systems, USA), 20 ng/ml human recombinant basic fibroblast growth factor (hrbFGF; R&D Systems), and 100 ng/ml heparin sodium salt (Sigma-Aldrich, USA). Media were changed every 24 h and all cells were cultured in humidified conditions with 5% CO_2_ at 37°C. EpiSC-like pESCs were sub cultured every 5–7 days using pulled glass pipettes. Expanded colonies were detached from the feeder cells and dissociated into small clumps. These clumps were transferred into new feeder cells containing mitomycin-C-treated (Roche, Germany) MEFs.

### Reverse Transcription-polymerase Chain Reaction (RT-PCR)

Total RNA was extracted from the cells using the TRIzol® Reagent (Invitrogen, USA) according to the manufacturer’s instructions. cDNA was synthesized using the High Capacity RNA-to-cDNA Kit (Applied Biosystems, USA) at 37°C for 1 h. Derived cDNA samples were amplified with 2× PCR master mix solution (iNtRON, Korea) and 2 pmol primers as shown in Table 1. PCR reactions were performed in a thermocycler under the following conditions: 94°C for 5 min, 35 cycles of denaturation at 95°C for 30 s, annealing for 30 s (annealing temperatures depended on each primer set), extension at 72°C for 30 s, and a final extension at 72°C for 7 min. Amplified PCR products were visualized using electrophoresis on 1% agarose gel stained with ethidium bromide.

**Table 1:** Primer sets for RT-PCR.

Gene	Primer sequence	Annealingtemperature (°C)	Productsize (bp)	Accessionnumber
*OCT4*	5′-AACGATCAAGCAGTGACTATTCG-3′	60	153	AF074419
	5′-GAGTACAGGGTGGTGAAGTGAGG-3′			
*NANOG*	5′-AATCTTCACCAATGCCTGAG-3′	60	141	DQ447201
	5′-GGCTGTCCTGAATAAGCAGA-3′			
*SOX2*	5′-CAACTCTACTGCTGCGGCG-3′	56	317	EU519824
	5′-CGGGCAGTGTGTACTTATCCTTC-3′			
*CRABP2*	5′-CTGACCATGACGGCAGATGA-3′	60	185	NM001164509
	5′-CCCCAGAAGTGACCGAAGTG-3′			
*DES*	5′-CCTCAACTTCCGAGAAACAAGC-3′	60	108	NM001001535
	5′-TCACTGACGACCTCCCCATC-3′			
*AFP*	5′-CGCGTTTCTGGTTGCTTACAC-3′	60	483	NM214317
	5′-ACTTCTTGCTCTTGGCCTTGG-3′			
*EGFP*	5′-GCGACGTAAACGGCCACAAGTTC-3′	60	599	YP003162718
	5′-GACCATGTGATCGCGCTTCTCG-3′			
*PSIP1*	5′-CTCCTCCCTGGGCTTCGGAC-3′	60	114	XM001927571
	5′-CTCGAGCTGGCCAATGAGGAT-3′			
*ß-ACTIN*	5′-GTGGACATCAGGAAGGACCTCTA-3′	60	137	U07786
	5′-ATGATCTTGATCTTCATGGTGCT-3′			

### Immunocytochemistry (ICC) and Alkaline Phosphatase (AP) Staining

ICC and AP staining were performed to evaluate expression of genes related to pluripotency and AP activity. Before staining, all cell samples were preincubated for 10 min at 4°C and fixed with 4% paraformaldehyde for 30 min. After washing twice with Dulbecco’s phosphate-buffered saline (DPBS; Welgene), samples were treated for 1 h with 10% goat serum in DPBS to blocking nonspecific binding. Serum-treated cells were incubated overnight at 4°C with the primary antibodies. The primary antibodies used were as follows: Oct4 (1∶100; Santa Cruz Biotechnology, USA), Sox2 (1∶100; Millipore, USA), Nanog (1∶100; Santa Cruz Biotechnology), SSEA4 (1∶100; Millipore), Tra-1-60 (1∶100; Millipore), and Tra-1-81 (1∶100, Millipore). When we used the antibodies for intracellular proteins such as Oct4, Sox2, and Nanog, fixed cells were treated for 5 min with 0.2% Triton-X100 (Sigma-Aldrich) before the serum blocking. After incubation with the primary antibody, the cells were treated for 3 h at room temperature with Alexa Fluor-conjugated secondary antibodies. Nuclei were stained with Hoescht 33342 (Molecular Probes, USA) or propidium iodide (PI; Sigma-Aldrich). Images of stained cells were captured using a LSM 700 Laser Scanning Microscope (Carl Zeiss, Germany) and processed with the ZEN 2009 Light Edition program (Carl Zeiss).

For AP staining, fixed EpiSC-like pESCs were incubated for 30 min at room temperature in the dark with 2% nitro blue tetrazolium chloride (NBT)/5-bromo-4-chloro-3-indolylphosphate toluidine salt (BCIP) stock solution (Roche) diluted in buffer solution (0.1 M Tris-HCl, 0.1 M NaCl, pH 9.5). Cells were then examined under an inverted microscope.

### Embryoid Body (EB) Formation and in vitro Differentiation

To evaluate the ability of *in vitro* differentiation, embryoid bodies were generated from EpiSC-like pESCs. Cultured embryonic stem cell colonies were detached from feeder cells, and colonies were mechanically dissociated into small clumps. Suspension cultures of these clumps were obtained using the hanging-drop method for 5–6 days with PESM in the absence of cytokines. After hanging-drop culture, small clumps were aggregated and formed embryoid bodies. Cultured embryoid bodies were seeded on 0.1% gelatin-coated plates and cultured for 2–3 weeks with DMEM containing 15% FBS. After 2–3 weeks, differentiated cells were fixed in 4% paraformaldehyde and analyzed using RT-PCR and immunostaining with differentiation-specific antibodies: neurofilament (ectoderm; 1∶100; Millipore), vimentin (mesoderm; 1∶100; Millipore), and cytokeratin 17 (endoderm; 1∶100; Millipore) as described above.

### Karyotype Analyses

Standard G-banding chromosome and cytogenetic analyses were used to karyotype the cell lines. Karyotyping was performed at Samkwang Medical Laboratories (Korea, http://www.smlab.co.kr/).

### Lentiviral Vector Production

Lentiviral vectors containing enhanced green fluorescent protein (EGFP) were produced as previously described [35] with some modifications. HEK 293 LTV cells (Cell Biolabs, USA) were used as the packaging cell line and cultured according to the manufacturer’s instructions. Four plasmids were used for the production of lentiviral vectors: self-inactivating lentiviral vector plasmid, pLL3.7; packaging plasmids, pLP1 and pLP2; and envelope plasmid, pLP/VSVG(Invitrogen). These plasmids were transfected into HEK 293 LTV cells using the calcium phosphate precipitation method. Two hours before transfection, the cells were incubated with 25 µM chloroquine (Sigma-Aldrich). After 12 h of transfection, transfected cells were treated with 15% glycerol solution for 90 s and cultured for another 24 h. Culture supernatants were harvested four times (every 12 h) and stored at 4°C. Harvested supernatants were filtered using 0.45-µm pore filters (Nalgene, USA) and concentrated by centrifugation at 18,000×*g* for 5 h at 4°C. The virus pellet was dissolved in PESM and stored at –76°C until use. The viral titer was calculated using the serial dilution method.

### Lentiviral Transgene Transduction and Flow Cytometric Analyses

Three to four days after passaging into new feeder cells, EpiSC-like pESCs were transduced with lentiviral vectors under various multiplicities of infection (MOIs) of 1–100. Transductions were performed for 24 h in PESM containing 8 µg/ml polybrene (Sigma-Aldrich) and concentrated virus. These transduced EpiSC-like pESCs were cultured in PESM without virus for another 4–5 days and then passaged. The parts of them were analyzed by flow cytometry. To analyse the EGFP expression level in transduced EpiSC-like pESCs under each MOI, EpiSC-like pESC colonies were detached from feeder layers and dissociated into single cells using TrypLE™ Express (Gibco) and fixed with paraformaldehyde. Fixed cells were analyzed using flow cytometery (FACSCalibur) and Cell Quest software (Becton Dickinson, USA).The data were processed using the software FlowJo (Tree Star Inc., USA).

### Genome Methylation Assay

To analyze methylation patterns in CMV promoter regions of lentiviral transgenes, genomic DNA of transduced cells was analyzed by bisulfite sequencing. First, genomic DNA was extracted using the G-spin™ Genomic DNA Extraction Kit for Cell/Tissue (iNtRON) and bisulfite treatment was performed using the EZ DNA Methylation-Gold™ Kit (Zymo Research, USA). Bisulfite-treated DNA samples were PCR-amplified with specific primers for the CMV promoter region (Table 2). Amplifications were performed using 2× PCR master mix solution and 2 pmol of primers designed using the Methprimer program (http://www.urogene.org/methprimer/index1.html). The resulting PCR products were separated by electrophoresis and purified from agarose gels using the MEGA-spin™ Agarose Gel Extraction Kit (iNtRON). Purified amplicons were cloned into the pGEMT-Easy Vector (Promega, USA) and transformed into *Escherichia coli* (DH5-α; Novagen, USA). Positive colonies were selected and plasmids were extracted from the selected colonies using the DNA-spin™ Plasmid DNA Purification Kit (iNtRON). The extracted plasmids were sequenced using an ABI PRISM 3730 automated sequencer (Applied Biosystems). Finally, the conversion rate of cytosine to thymine was calculated to be more than 99%, and converted sequences were processed using the BIQ Analyzer Program (http://biq-analyzer.bioinf.mpi-inf.mpg.de/) and the original sequences to analyze the methylation patterns.

**Table 2:** Primer sets for bisulfite sequencing.

	Primer sequence	CpG	Annealingtemperature (°C)	Productsize (bp)
CMV	5′-ATGATTTTATGGGATTTTTTTATTTG-3′	14	56	279
	5′-ATTCACTAAACCAACTCTACTTATATAAAC-3′			
EGFP	5′- TGGGGTATAAGTTGGAGTATAATTATAATA-3′	18	54	259
	5′- AACTCCAACAAAACCATATAATC-3′			

### 5-Aza-2'-deoxycytidine (5-AzadC) and Trichostatin A (TSA) Treatments

Late-passage EGFP-transduced EpiSC-like pESCs were treated with inhibitors of repressive epigenetic markers to evaluate the relationship between viral transgene expression and epigenetic modifications. 5-Aza-2′-deoxycytidine (5-AzadC; Sigma-Aldrich) and trichostatin A (TSA; Sigma-Aldrich) (DNA methyl transferase and histone deacetylase inhibitors, respectively) were used based on previous reports [33], [36], with some modifications. Three to four days after passage into new feeder cells, EpiSC-like pESCs were treated with 100 nM 5-AzadC (for 48 h), 10 nM TSA [for the first 24 h and dimethyl sulfoxide (DMSO) only for the second 24 h), or DMSO (for 48 h; Edwards Life sciences, USA) as a vehicle-only control. After treatments, cells were cultured for 2 more days without inhibitors or DMSO. Finally, treated cells were analyzed using flow cytometry. Bisulfite sequencing was performed with genomic DNA samples extracted as previously described.

### Statistical Analyses

All efficiency data from flow cytometric analyses were statistically analyzed using the “R” program (http://www.r-project.org). Statistical significance between data was determined by one-way analysis of variance (ANOVA) and Tukey’s honestly significant difference (HSD) test. Differences were considered significant when the *P*-value was less than 0.05.

## Results

### Establishment and Characterization of Epiblast Stem Cell-like Porcine Embryonic Stem Cells

**Figure 1:**
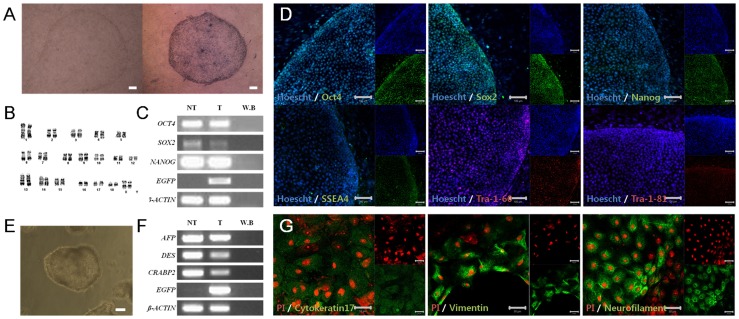
Derivation and characterization of EpiSC-like pESCs. (A) EpiSC-like pESCs derived from *in vitro*-produced embryos represented typical morphologies of mouse epiblast stem cells and human embryonic stem cells, and have alkaline phosphatase activity (left panel: no-stained colony; right panel: AP stained colony). (B) EpiSC-like pESCs have a normal karyotype (36+ XX) and (C) expressed genes related to pluripotency, as determined by RT-PCR (NT: non-transfected EpiSC-like pESCs passage 14, T: transfected EpiSC-like pESCs passage 14, W.B.: water blank). (D) Expression of pluripotent markers was detected using immunocytochemistry (passage number: 13). (E) Embryoid bodies were generated in suspension culture and spontaneously differentiated onto culture dishes. (F, G) The differentiated cells expressed differentiation marker genes at the mRNA and protein levels (Ectoderm: *CRABP2*, neurofilament, Mesoderm: *DES*, vimentin, Endoderm: *AFP*, cytokeratin 17; passage number: 17). Scale bars = 100 µm, except for A and G (scale bar = A: 200 µm, G: 50 µm).

First, the pluripotent cell line used in this study was generated from porcine preimplantation embryos. *In vitro* produced blastocysts were used for the derivation of EpiSC-like pESCs as previously described [8], [37]. Two EpiSC-like pESC lines were established from 42 hatched blastocysts (Derivation efficiency: 4.78% (2/42)). Of these two established cell lines, only one was used for further studies. Established EpiSC-like pESCs are represented by typical flattened morphologies as previously reported [8], similar to mEpiSCs and human embryonic stem cells. Additionally, they possess AP activity (Fig. 1A) and are stably maintained over long periods (>50 passages in 1 year) with a normal karyotype (Fig. 1B; 36+ XX). These cells were analyzed for pluripotent marker expression and their differentiation ability *in vitro* to verify their pluripotency, according to previously reported standards [8]. Expression of pluripotency-related transcription factors such as *OCT4*, *SOX2*, and *NANOG* were detected at the mRNA level (Fig. 1C). These factors, as well as EpiSC-like pESC surface markers such as SSEA4, TRA-1-60, and TRA-1-81, were also identified at the protein level using immunocytochemistry (Fig. 1D). When these cells were detached from feeder cells and cultured in suspension, they aggregated and subsequently formed embryoid bodies (EBs; Fig. 1E). The generated EBs spontaneously differentiated into three germ layers upon being placed on gelatin-coated plates. In the differentiated cells, the expression of differentiation marker genes on the three germ layers was detected by RT-PCR and immunostaining (ectoderm: *CRABP*, neurofilament, mesoderm: *DES*, vimentin, endoderm: *AFP*, cytokeratin 17; Fig. 1F, G). Thus, we confirmed that the established cell line was a pluripotent cell line with the differentiation potential to generate the three germ layers.

### Lentiviral Transgene Introduction into Epiblast Stem Cell-like Porcine Embryonic Stem Cells

To deliver an exogenous gene into the EpiSC-like pESCs, lentiviral vectors were employed as a carrier. A lentiviral vector containing EGFP as a reporter was used to investigate the introduction of lentiviral transgenes into EpiSC-like pESCs. In a previous report, transgenic mouse and mouse ESCs were successfully produced using this lentiviral construct [38]. The estimated titer of produced lentiviral vectors in 293 LTV cells was approximately 9×10^9^ viral particles/ml. Using these vectors, EpiSC-like pESCs were transduced for 24 h under several MOIs (1, 5, 10, 25, 50, 75, and 100). Unlike somatic cells, EpiSC-like pESCs lose their characteristics, including typical morphology and pluripotency, when cultured without feeder cells. Therefore, EpiSC-like pESCs were transfected in culture on feeder cells and MOIs were calculated based on the number of MEFs and EpiSC-like pESCs.

**Figure 2:**
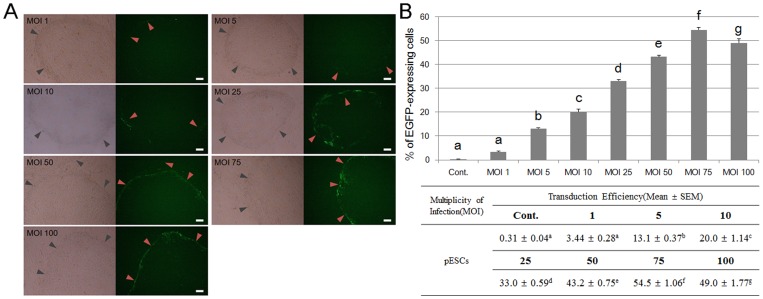
Lentiviral transduction of EpiSC-like pESCs. (A) Lentiviral-transduced EpiSC-like pESCs with several MOIs were cultured stably and passaged (left panels: bright field, right panels: EGFP, gray arrows: boundary of colonies, red arrows: EGFP-expressing cells; scale bar = 100 µm). (B) The transduced cells have different expression levels depending on MOI. Efficiency was measured using flow cytometry (mean ± S.E.M, *n* = 3). Values noted by a–h indicate they are significantly different.

**Figure 3:**
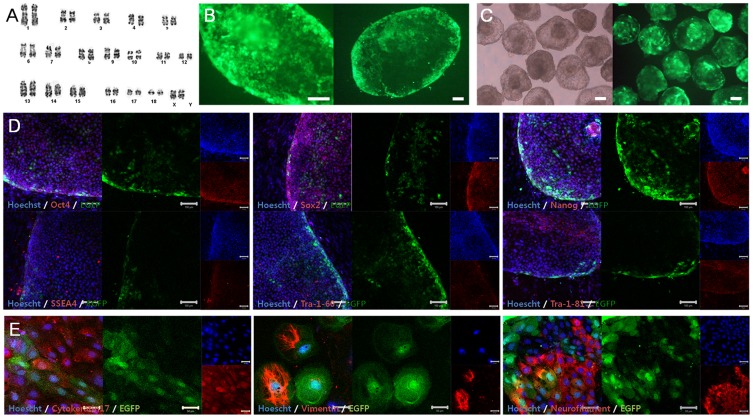
Characterization of EGFP-transduced EpiSC-like pESCs. (A) EGFP-transduced EpiSC-like pESCs have a normal karyotype (36+ XX). (B) The proportion of EGFP-expressing cells increased to 70–80% via selection for the EGFP-expressing part of colonies. (C) Embryoid bodies formed from EGFP-expressing EpiSC-like pESCs and expressed EGFP after aggregation (left panel: bright field, right panel: EGFP; scale bar = 100 µm). (D) EGFP-expressing EpiSC-like pESCs expressed pluripotent genes at the protein level (scale bar = 100 µm; passage number: 13) and (E) could be differentiated into three germ layers (scale bar = 50 µm; passage number: 23).

EGFP was successfully introduced into the cells via lentiviral vectors under various MOIs. EGFP expression was detected in the cells under an inverted microscope, although expression differences existed depending on the MOI (Fig. 2A). The EGFP expression levels quantified by flow cytometry significantly increased up to a MOI of 75 in a dose-dependent manner but decreased by approximately 5% at a MOI of 100 (Fig. 2B). EGFP expression was heterogeneous in the colony of cultured EpiSC-like pESCs, particularly concentrated on part of the boundary. Therefore, it is possible that expression levels could be increased up to 70–80% in a single colony by selecting the EGFP-expressing part of colonies during subculture (Fig. 3B). Cell characteristics such as viability and proliferation were rarely affected, except at a MOI of 100 in which the cells exhibited cellular toxicity post-transduction.

EGFP-transduced EpiSC-like pESCs were characterized to assess whether pluripotency was affected by transduction. Transduced EpiSC-like pESCs under a MOI of 75 possessing the highest expression levels were used for characterization. Transduced EpiSC-like pESCs could be stably maintained over an extended time period (>50 passages) using the same general EpiSC-like pESC culture methods and had an identical karyotype (36+ XX) as before transfection (Fig. 3A). EGFP expression, as well as transcription factors related to pluripotency, was detected in transfected cells at the mRNA and protein levels as measured by RT-PCR and immunostaining, respectively (Figs. 1C and 3D). These cells could be differentiated into three germ layers *in vitro* as determined by EB formation (Fig. 3C, E). In brief, transduction-induced abnormalities, such as physiological features and pluripotency post-transfection, were not detected in EGFP-transduced EpiSC-like pESCs.

### Decreased EGFP Expression with Extended Culture and Recovery of EGFP Expression by Differentiation

**Figure 4:**
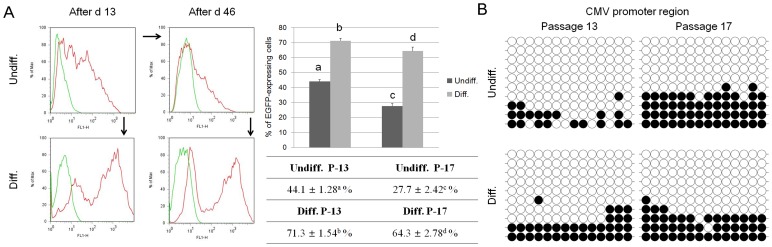
Change in EGFP expression during extended culture and *in vitro* differentiation. (A) EGFP expression levels of transduced EpiSC-like pESCs declined during extended culture. The decreased expression recovered during *in vitro* differentiation. (B) To analyze the effects of the epigenetic state on transgene expression, the DNA methylation patterns of the CMV promoter region in the transgene of each sample were evaluated via bisulfite sequencing. Along with decreases in EGFP expression, the DNA methylation levels of the CMV promoter region increased. Although DNA methylation levels increased during extended culture, they were not altered by *in vitro* differentiation. (Each circle indicates individual CpG dinucleotides. White and dark circles represent unmethylated and methylated CpGs, respectively. Each row represents one individual clone of amplified PCR products). Values noted by a–h indicate they are significantly different. Data are the mean± S.E.M. (*n* = 3).

The proportion of cells expressing EGFP decreases when cultured for an extended time period without selection. Therefore, additional transfections were attempted to investigate this phenomenon. Thirteen days after transduction, using EpiSC-like pESCs (passage 12) under a MOI of 75, the proportion of cells expressing EGFP as measured by flow cytometry was 44.1±1.28% (Fig. 4A). Decreased EGFP expression was observed to be approximately 10% compared with the results of previous transduction (54.5±1.84% of cells expressed EGFP) (Fig. 2B). Furthermore, when cultured for longer than 13 days without selection, EGFP expression level decreased to 27.7±2.42% after 46 days. Unexpectedly, however, the decreased EGFP expression in transduced EpiSC-like pESCs was recovered by spontaneous *in vitro* differentiation. EGFP expression increased from 54.5% and 27.7% to 71.3% and 64.3% in the respective samples as measured by flow cytometry analyses. Note that EGFP expression recovered during differentiation, and the recovered EGFP expression levels of differentiated cells were higher than that of EpiSC-like pESCs.

Therefore, we hypothesized that these phenomena, including changes in EGFP expression due to extended culture and differentiation, were caused by epigenetic modifications and cellular status. First, to identify the effect of the cellular state on the expression of transgenes, MEFs and PEFs as somatic cell controls were transduced. Compared to transduced EpiSC-like pESCs, the rate of cells expressing EGFP in MEFs or PEFs was 81.2±0.70% and 74.8±5.71%, respectively, at a MOI of 75, which is similar to the expression level of differentiated cells from EpiSC-like pESCs (Fig. S2).

To analyze the effects of the epigenetic state among cell types on transgene expression, the DNA methylation patterns of the CMV promoter region in the transgene of each sample (Fig. 4A) were evaluated via bisulfite sequencing. During extended culture, methylation levels in the CMV region increased as the EGFP expression decreased (Fig. 4B). Cells from early passages have much lower methylation levels compared to those from late passage cells. Additionally, early passage cells had irregularly scattered patterns in terms of methylation sites. However, when spontaneously differentiated, the degree of DNA methylation did not change, but GFP expression increased during differentiation. The CMV promoters of differentiated cells were methylated similar to those of non-differentiated cells (Fig. 4B). When transfected somatic cells such as MEFs and PEFs were analyzed, the methylation pattern of the CMV promoter was completely unmethylated (Fig. S3).

### Decreased EGFP Expression during Extended Culture due to DNA Methylation of Promoter Regions

**Figure 5:**
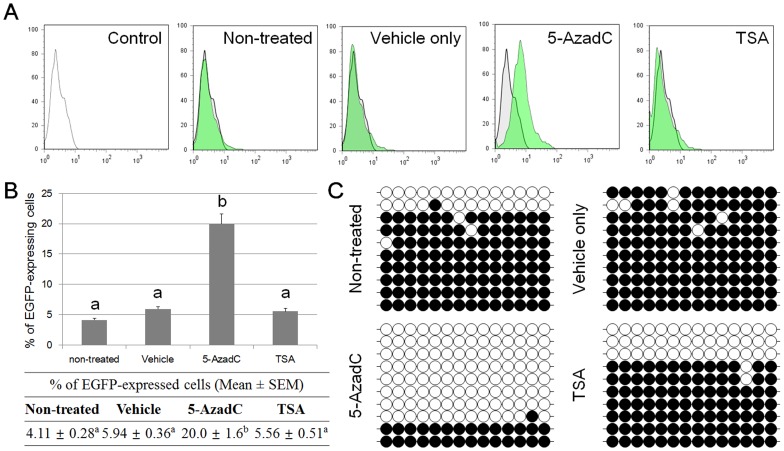
Silenced transgenes can be reactivated by treatment with the DNA methylase inhibitor, 5′-aza-2′-deoxycitidine (5-AzadC). (A) 5-AzadC treatment reactivates the silenced transgene, EGFP. (B) The recovered expression level was measured by flow cytometry. (C) 5-AzadC demethylated the methylated CMV promoter region. Values noted by a–h indicate they are significantly different. Data are the means± S.E.M. (*n* = 3).

To evaluate the effect of methylation on EGFP expression during extended culture, EpiSC-like pESCs were treated with inhibitors of repressive epigenetic marks. Control and experimental groups consisted of four: non-treated, DMSO-, 5-AzadC-, and TSA-treated samples. Late passage (passage 46) EpiSC-like pESCs expressed low levels of EGFP (4.11±0.28%). Samples treated with DMSO and TSA did not show a recovery of EGFP expression and showed similar expression levels as nontreated samples. Although these two groups did not affect expression of the transgenes, the silenced EGFP expression recovered to 20.0±1.6% upon treatment with 5-AzadC (Fig. 5A, B). The effect of 5-AzadC on the expression of transgenes was determined via bisulfite sequencing. Among the four groups, CMV promoter region demethylation occurred after 5-AzadC treatment (Fig. 5C).

## Discussion

Because pluripotent cells have great potential as a cell source, research in this area has focused on embryonic carcinoma cells to iPSCs [4], [39]. Embryonic stem cell research began in 1981 by the establishment of mouse embryonic stem cells and was accelerated by the establishment of human embryonic stem cells and iPSCs in 1998 and 2006, respectively [1], [4], [40]. The purpose of pluripotent stem cell research in humans and mice includes the elucidation of basic cellular mechanisms contributing to the maintenance of pluripotency. In humans, applications exist in cell therapies. However, in domestic animals, the research aim is to create an indefinite cell source for transgenic animals used as bioreactors and tissue engineering materials as well as preliminary studies for human research [9]. Because of the physiological and immunological similarities that exist between pigs and humans, porcine pluripotent cell lines have been identified as important candidates for preliminary studies on human disease as well as a source for generating transgenic animals [10]–[12]. Therefore, the establishment and characterization of pESCs, along with the generation of stable transgenic cell lines, are vitally important. In our previous studies, we developed a reproducible method for the establishment of EpiSC-like pESCs and iPSCs and determined that similar to humans, pig is a nonpermissive species and pig pluripotent stem cells show a primed pluripotent state regarding marker gene expression patterns and X chromosome inactivation [8], [16].

To achieve such goals, stem cell engineering involving the introduction of transgenes into cells has been developed. In mice and humans, many transfection methods, including viral vectors, liposome-mediated gene delivery, and electroporation, have been studied [20]–[23]. In the porcine model, however, much fewer studies have been reported. In the first reported study, EGFP-transduced embryonic germ cells (EGCs) were established by the introduction of plasmid vectors using liposomal vectors [30]. In a second study, plasmid vectors containing humanized renilla green fluorescent protein (hrGFP) were introduced into pESCs was via electroporation [17]. Although GFP-expressing pESC lines were established via electroporation, transfection efficiency was very low (only three stably GFP-expression lines from 12 trials), and a GFP-expressing line was not obtained by retroviral and liposome-mediated transfection. Therefore, we undertook our study to develop a system for efficient introduction of transgenes into embryo-derived pluripotent stem cells. Our previous studies showed that EpiSC-like pESCs have very high cytotoxicity when treated with liposome-mediated and electroporation methods, as well as low colony-forming rates from dissociated single cells, similar to humans [8], [41]. Therefore, viral vectors were selected because plasmid vectors are not suitable for generating a stable transgene expression cell line. And because lentiviral vectors can infect dividing cells as well as nondividing cells, lentiviral vectors were chosen for transfection of EpiSC-like pESCs having long doubling times of approximately 36 h (Fig. S1), similar to human ESCs [7], [27].

Historically, although retro- and lentiviral vectors have been widely used for the production of transgenic animals and the establishment of transgenic pluripotent stem cells, silencing of the viral transgenes caused by epigenetic modifications and *trans*-acting factors remains an obstacle to be resolved [31]–[34], [42]–[44]. To overcome these silencing problems in cells, particularly embryos and pluripotent cells, various approaches have been developed. The major difficulties post-transfection, the repression of long terminal repeats (LTRs) in the viral genome known as transcriptional regulator by *de novo* DNA methylation and repressive *trans*-acting factors [45], [46], were resolved using bidirectional or internal promoters such as the CMV promoter, which is strongly expressed in various tissues, [47], [48] and the deletion or modification of LTR sequences to prevent recruitment of repressive *trans*-acting factors [43], [49]. Using regulatory elements, including woodchuck hepatitis virus response element (WRE) [50], HIV FLAP [51], and matrix attachment region (MAR) [52], which are responsible for transcript stabilization and the translocation of provirus into nuclear and DNA loop formation, respectively, also improve transgene expression.

Prior to transfection, we established a new EpiSC-like pESC line using the whole seeding method, as previously reported [8]. The established line exhibited the general characteristics of a primed pluripotent state in terms of marker gene expression, ability to differentiate *in vitro*, and normal karyotype (Fig. 1). The lentiviral construct used in this study is pLL3.7 consisted of EGFP as a marker gene with the CMV promoter and *cis*-acting elements including FLAP, WRE, 3′SIN-LTR and multiple cloning site for shRNA. The reason this construct was chosen is as follows: 1) This vector has been proven to work efficiently in mouse embryos, primary cells and embryonic stem cell lines, and transgenic mice have been generated using mouse ESCs and embryos transfected with this vector [38]. 2) In previous transfection studies involving porcine pluripotent cells, the CMV promoter was used for the establishment of transgene-expressed cell lines, these resulting transduced cell lines stably expressed the transgene for more than 90 months [17], [30]. When EpiSC-like pESCs were transfected with this vector under various MOIs, cytotoxicity was not detected up to a MOI of 75. At a MOI of 100, however, cytotoxicity occurred and reduced the number of EGFP-expressing cells (Fig. 2B). Moreover, EGFP expression was stronger at the edge of the colonies (Fig. 2A), likely because of metabolic upregulation and high cell density due to proliferation of dividing cells at the edge of the colonies.

To examine the decline in EGFP expression during extended culture, we assessed whether transgene expression was affected by DNA methylation or histone acetylation, which are known to epigenetically regulate gene expression. Methylated cytosine in DNA represses gene expression via recruitment of proteins associated with heterochromatin such as MeCP2. Acetylation on histone tails activates gene expression by increasing the negative charge of histones [44]. Bisulfite sequencing data indicated that the DNA methylation level of the promoter region and expression of the transgene were negatively correlated because DNA methylation in the CMV promoter region increased with a concomitant decrease in EGFP expression (Fig. 4). However, methylation levels of the EGFP region were not related to transgene expression (data not shown), in contrast to a previous report involving transgenic animals [33]. The EGFP region was hypomethylated regardless of EGFP expression.

The expression level of transgenes is dependent upon the vector construct, transfection methods used and cell types. It is therefore important that the characterization of vector activities in various cell types is given attention. In the case of the CMV promoters used in this study, differences in expression patterns have been reported in several papers via the characterization of the transcriptional activities in ESCs. Some studies showed that the CMV promoter is active in human or mouse ESCs during long term culture [38], [53]–[55]. Conversely, other studies have reported that CMV-driven transgene expressions are rapidly downregulated or inactive in human or mouse ESCs. Silenced transgenes were not reactivated during differentiation [56]–[59]. In porcine studies, CMV-driven expression of GFP was stably expressed during the propagation of pESCs (over 20 months) [17], and transgenic porcine embryonic germ cell lines were successfully established using a CMV-EGFP construct [30]. Our data has shown that the CMV-driven expression of GFP was progressively downregulated by DNA methylations and reactivated during differentiation by transacting factors without changes of DNA methylation levels. This result is consistent with previous findings of the latter cases in humans and mice with the exception of the upregulation of transgenes during differentiations. The differences of our results with previous porcine studies may be as a result of the different vector construct and transfection method used. The reactivation of silenced transgenes during differentiation that we observed in this study indicate that trans-acting factors, as well as DNA methylations, affect transgene silencing in undifferentiated EpiSC-like pESCs.

When transduced cells were treated with 5-AzadC and TSA, inhibitors of DNA methylation and histone deacetylases, respectively, 5-AzadC allowed reactivation of silenced expression, but TSA did not (Fig. 5A, B). Bisulfite sequencing of the promoter regions showed that 5-AzadC-treated samples were hypomethylated in CMV promoter regions compared with the other treated groups, which had hypermethylated promoters. These results clearly demonstrate that decreased transgene expression is related to DNA methylation in promoter regions, not histone modifications.

In addition to silencing of gene expression during extended culture, the transfection efficiency of EpiSC-like pESCs was lower than that of other embryo-originated somatic cells (Fig. S2). However, PSIP1, a protein that participates in lentiviral provirus integration into host genomes [60], was examined by RT-PCR and no difference was observed among EpiSC-like pESCs, PEFs, and MEFs (Fig. S4). This result suggests that the low efficiency of transfection is not related to the low integrity of the provirus. In addition, because the copy number of an inserted lentiviral construct could affect EGFP expression, a correlation between EGFP expression level and transgene copy number was verified. However, because of heterogeneity in the transduced cells due to the random insertion of multiple copies, it is hard to quantitatively measure the copy number of inserted transgenes. So, transgene copy number was relatively quantified by real-time RCR. Real-time PCR results showed that changes in EGFP expression and transgene copy number are not correlated (Fig. S5). And also reactivation of silenced transgenes during differentiation clearly demonstrated that low expression levels are not due to low integrity (Fig. 4A). Note that DNA methylation levels of the CMV promoter region did not change during differentiation although transgene expression increased. Undifferentiated and differentiated cells at the same passage have similar levels of DNA methylation in the CMV promoter (Fig. 4B). This suggests that the recruitment of transcription factors in differentiated cells or the downregulation of repressive *trans*-acting factors, rather than demethylation of the promoter region, is involved in the unmethylated open chromatin for reactivation of silenced transgenes. In fact, many binding sites for putative transacting factors such as cyclic AMP-response elements (CRE), NF-Kappa B, AP-1, serum response elements exist in the CMV promoter [61]. These sites could be predicted by the program TFSEARCH ver. 1.3 (http://www.cbrc.jp/research/db/TFSEARCH.html). This mechanism is supported by the phenomenon that more methylated cells in late passages showed lower reactivation of transgenes (Fig. 4A). Therefore, low transgene expression in pluripotent cells is likely because of *trans*-acting factors as well as DNA methylation.

In conclusion, we were able to successfully derive EpiSC-like pESCs and introduced an EGFP transgene into these cells using lentiviral vectors. Transgene expression in EpiSC-like pESCs was altered during maintenance and differentiation due to epigenetic changes. Although we used modified lentiviral vectors containing regulatory elements, we could not prevent the transgene silencing that occurred due to DNA methylation and *trans*-acting factors. Nonetheless the silenced transgene expression was reactivated by differentiation and treatment of 5-AzadC. Therefore, this system could be applied for the short term analysis of gene function in EpiSC-like pESCs, induction of differentiation, tracking of transplanted cells and the production of chimeras. Finally, this cell line could potentially be used as a donor cell source for transgenic pigs and serve as a useful tool for studies involving pESCs and gene therapy in humans, as well as aid in the understanding of epigenetic regulation of transgenes.

## Supporting Information

Figure S1 **Doubling time of epiblast stem cell-like porcine embryonic stem cells.** Because EpiSC-like pESCs have long doubling times of approximately 36 h, similar to human ESCs, lentiviral vectors were chosen for transfection of EpiSC-like pESCs.
(TIF)

Figure S2 **Lentiviral transduction efficiency of MEFs and PEFs at various MOIs.** MEFs and PEFs were transfected at various MOIs for comparing with EpiSC-like pESCs. The transfection efficiency of embryo-originated somatic cells was higher than that of EpiSC-like pESCs. The EGFP expression levels quantified by flow cytometry significantly increased up to a MOI of 50 in a dose-dependent manner and were reached a plateau from MOI of 75.
(TIF)

Figure S3 **Methylation level of the CMV promoter region in MEFs and PEFs.** To compare the DNA methylation patterns of the CMV promoter region between transfected porcine pluripotent cells and somatic cells such as MEFs and PEFs, bisulfite sequencing was performed. The CpGs were completely unmethylated in CMV promoter of MEFs and PEFs.
(TIF)

Figure S4 **Expression level of** ***PSIP1*** **in MEFs, PEFs, and EpiSC-like pESCs.** PSIP1, a protein that participates in lentiviral provirus integration into host genomes, was examined by RT-PCR and no difference was observed among EpiSC-like pESCs, PEFs, and MEFs.
(TIF)

Figure S5 **Correlation between the copy number of inserted transgenes and the EGFP expression level.** To analyze the effects of transgene copy number on changes of transgene expression, the transgene copy number was relatively quantified by real-time RCR. Real-time PCR results showed that changes in EGFP expression and transgene copy number are not correlated.
(TIF)

Table S1 **Lentiviral transduction efficiency in PEFs and MEFs.**
(DOCX)
