# Supplementary material for: Epigenetic Changes of Lentiviral Transgenes in Porcine Stem Cells Derived from Embryonic Origin
Source: PLoS One. 2013 Aug 19;8(8):e72184. doi: 10.1371/journal.pone.0072184 (PMC3747048; doi:10.1371/journal.pone.0072184)
Supplement: Table S1 — Lentiviral transduction efficiency in PEFs and MEFs. (DOCX) [file pone.0072184.s006.docx]

**Table S1.** Lentiviral transduction efficiency in PEFs and MEFs

| Multiplicity of Infection(MOI) | Transduction Efficiency(mean ± SD) | | | | | | | |
| --- | --- | --- | --- | --- | --- | --- | --- | --- |
|  | **Cont.** | **1** | **5** | **10** | **25** | **50** | **75** | **100** |
| PEF | 0.11 ± 0.04^a^ | 1.34 ± 0.17^a^ | 11.7 ± 0.83^b^ | 13.6 ± 0.96^b^ | 54.5 ± 2.43^c^ | 68.7 ± 6.29^df^ | 74.8 ± 4.49^dfh^ | 76.1 ± 5.71^dg^ |
| MEF | 0.22 ± 0.07^a^ | 3.57 ± 0.19^a^ | 22.6 ± 1.15^e^ | 30.1 ± 0.85^e^ | 67.0 ± 0.69^f^ | 79.8 ± 1.03^gh^ | 81.2 ± 0.70^gh^ | 82.8 ± 0.93^g^ |

*Values denoted by a–h are significantly different.
